# “If we can show that we are helping adolescents to understand themselves, their feelings and their needs, then we are doing [a] valuable job”: counselling young people on sexual health in the Brook Advisory Centre (1965–1985)

**DOI:** 10.1136/medhum-2021-012206

**Published:** 2021-08-23

**Authors:** Caroline Rusterholz

**Affiliations:** History, University of Cambridge, Cambridge, UK

**Keywords:** history, medical humanities, sexual medicine

## Abstract

First opened in 1964 in London, the Brook Advisory Centres (BAC) were the first centres to provide contraceptive advice and sexual counselling to unmarried people in postwar Britain. Drawing on archival materials, medical articles published by BAC members and oral history interviews with former counsellors, this paper looks at tensions present in sexual health counselling work between progressive views on young people’s sexuality and moral conservatism. In so doing, this paper makes two inter-related arguments. First, I argue that BAC doctors, counsellors and social workers simultaneously tried to adopt a non-judgmental listening approach to young people’s sexual needs and encouraged a model of heteronormative sexual behaviours that was class-based and racialised. Second, I argue that emotional labour was central in BAC staff’s attempt to navigate and smooth these tensions. This emotional labour and the tensions within it is best illustrated by BAC’s pyschosexual counselling services, which on the one hand tried to encourage youth sexual pleasure and on the other taught distinctive gendered sexual roles that contributed to pathologising teenage sexual behaviours and desire.

In all, I contend that, in resorting to an emotionally orientated counselling, BAC members reconfigured for the young the new form of sexual subjectivity that had been in the making since the interwar years, that is, the fact that individuals regarded themselves as sexual beings and expressed feelings and anxieties about sex. BAC’s counselling work was as much a rupture with the interwar contraceptive counselling tradition—since it operated in a new climate, stressed a non-judgmental listening approach and catered for the young—as it was a continuity of some of the values of the earlier movement.

In 1965, in the London neighbourhood of Fitzrovia, close to many universities, an unmarried ‘girl’ aged 21 years walked through the door of the Brook Advisory Centre (BAC), the first institution to officially provide contraceptive advice to young unmarried people in postwar Britain, which had opened in 1964. After a counselling session with a doctor, she walked out with contraceptives. The young woman told the doctor that she was not a virgin but had already had three ‘backstreet’ abortions. For the first time, the girl explained, she had fallen in love and this new relationship was meaningful to her. For this reason, she wanted to wait until she was married to have a family and sought birth control in the meantime, as ‘she could not afford to have a baby and would never be able to kill a baby of a man she loves’ (Brook 1966). Providing contraception to an unmarried girl in Britain was not entirely new in 1965; some family planning clinics unofficially advised young women provided that they showed proof of engagement, usually a (fake) ring. However, what made BAC distinctive was that, for the first time, a charity was providing contraception to young people openly and without subterfuge.

The history of BAC counselling is part of the longer British history of sexual counselling, and more specifically of contraceptive counselling. This history started back with the birth control movement. In the 1920s, the first birth control clinic opened in London, quickly followed by others across Britain. At first, such clinics were mainly concerned with providing information and prescribing birth control methods for married women. As they had to fight to be considered respectable and to dissociate contraception from its earlier association with free love, the leaders of the birth control movement underlined that their main goal was to encourage stable and loving marriages and babies that were wanted. This movement was aimed at helping married working-class mothers, overburdened by too many pregnancies, to limit and space births. Eugenic ideas about differential fertility were also part of the narrative supporting the birth control movement. In 1942, due to increasing demand from patients, some family planning centres expanded their remits to encompass psychosexual counselling and infertility counselling, which was the provision of advice on sexual disorders and therapies, and advice and treatments for infertility, respectively (Audrey 1980; Cohen 1993; Cook 2004; Davey 1988; Debenham 2014; Hall 1997; Rusterholz 2020). Infertility counselling was, however, already provided in Marie Stopes birth control clinics, a concurrent institution. This new orientation of the work done in Family Planning Association (FPA) centres was, however, slow to develop, facing opposition from many FPA medical professionals who did not see psychosexual work as being their responsibility and who considered those interested in this topic as a ‘lunatic fringe’ (Rusterholz 2020). Other organisations, such as the Marie Stopes Clinic, the Marriage Guidance Council and the Catholic Marriage Advisory Council to name a few, also started to provide sexual counselling for married couples in the early 1950s (Chettiar 2015; Harris 2015; Lewis, Clark, and Morgan 1992). However, it was only the opening of BAC that provided unmarried young people with a place to obtain contraception and advice on their sex lives. BAC opened as a preventive service to mitigate the risks engendered by the increase in premarital sex. In the postwar years, a new obsession with young people’s behaviour emerged. This was partly because young people were more numerous, which made them more visible due to the postwar baby boom (Jackson and Bartie 2014; Tebbutt 2016). Furthermore, in the late 1950s, young people’s sexual behaviour was starting to create growing anxieties among mass media and contemporary commentators (Cook 2004; Haste 1992; Rusterholz 2021a,Rusterholz 2021b). These anxieties were triggered by data showing an increase in the number of brides who were pregnant at marriage, the illegitimacy rate and the incidence of venereal diseases and backstreet abortions. Furthermore, it was felt that these illegitimacy rates were now also occurring in young women of a more privileged and better-educated social class (Dyhouse 2014; Hall 2013).

It was in this context that Helen Brook opened the BAC, the first charity helping unmarried people aged 16 (the age of consent) to 25 years. Born in 1907, Helen Brook was a mother of three, an artist and the wife of the economist Robin Brook; she had joined the FPA in the late 1940s, first working as a volunteer interviewer at the Islington clinic (Brook 1990). She then became chairman of the Islington clinic for 12 years. When the birth control activist Marie Stopes died in 1958, Brook was asked to reorganise and direct the Marie Stopes clinic in Whitfield Street, London. She took the opportunity to try a new experiment in one of the Stopes offices, hence avoiding the self-imposed limitation of the FPA service to married women and about-to-marry women. In 1962, she started to advise unmarried women (who were not even engaged) brought to her by social workers, and later set up ‘secret sessions’ for ‘girls and young men, often still at school or university’ (Marie Stopes Memorial Foundation 1963). Her motivation for holding such sessions, as she reflected 4 years after their creation in 1966, was to tackle the issue of rising rates of illegitimate births. She attributed this increase to several factors, namely the larger population of young people, earlier puberty, the greater freedom and mobility of the young and immigration from Ireland and what she called ‘backwards countries’ (Brook 1966). As these elements suggest, race and class were central in the creation of BAC, showing a continuity with the FPA’s emphasis on working-class women. Their main target clientele was young white women brought in by social workers, usually from deprived working-class backgrounds, who were deemed in need of monitoring, as well as white middle-class and upper-class young couples at university who were said to be in committed relationships ‘but unable to marry due to financial reasons’ (Marie Stopes Memorial Foundation 1964). These young couples were presented as in need of advice, rather than control, in order to avoid unwanted pregnancies that would jeopardise their future. Irish immigrants and those from ‘backwards countries’, most likely immigrants from the West Indies, South Asia and Africa based on postwar migration trends, were also potential clients. As many studies have shown, young black boys from the West Indies were usually presented by the mass media and social scientists and experts as sexual predators, allegedly bringing venereal diseases to Britain, while white working-class girls were always thought to be more promiscuous than white middle-class girls (Bingham 2009; Bivins 2015; Dyhouse 2014; Jackson and Bartie 2014; Webster 1998). Both groups were therefore perceived as vulnerable populations that needed to be educated in order to behave responsibly. This meant that, from the start, the white/middle-class norm of committed young people in steady relationships functioned as the model of ‘good’ sexual behaviour.

Following the opening of these sessions, the Marie Stopes board suggested that it would be advisable for Helen Brook to find a separate organisation. She received an anonymous donation of £5000 per year over 3 years to expand this work, and, as a result, the BAC officially opened in July 1964. Its opening officially condoned young people’s sexuality and recognised, for the first time, that young people needed to be protected from unwanted pregnancy. The provision of contraception to young, unmarried people took place before the 1967 Family Planning Act, which allowed but did not require local authorities to provide birth control to all women regardless of their marital status. Following the opening of the London branch, other BAC clinics were set up in locations such as Cambridge (1966), Birmingham (1966), Bristol (1968) and Edinburgh (1968). During the late 1960s and 1970s, the use of the service offered by BAC grew drastically and the demand quickly exceeded the capacity of the centres, resulting in long waiting lists. From 1056 clients in 1965, the number seen in BAC centres increased to 60 000 by 1979.

BAC played a pioneering role in counselling young people about their sexual reproductive health, and this is the first study that specifically examines the practical aspects of sexual health counselling. This article draws on archival materials from BAC available at the Wellcome Library; medical articles published by BAC members and four oral history interviews with former counsellors of BAC. These oral history interviews were conducted over the phone between March and May 2020. Oral and written consents were obtained and recorded prior to the interview for each participant. Participants agreed for their interview to be quoted and for their name to appear in this article.

By closely examining the content of sexual health counselling and medical guidance offered by BAC from its creation in 1964 to the setting up of a counselling Advisory Committee in 1988, which harmonised counselling practices across the centres (Brook Advisory Centre 1988), this article explores the tensions in BAC’s counselling work. First, I scrutinise what BAC members defined as the key components and values underpinning counselling. I argue that tensions were inherent in BAC’s counselling services; BAC doctors, counsellors and social workers tried to encourage a model of heteronormative sexual behaviours that was class-based and racialised—this model was based on the same type of principles that informed the family planning movement—while at the same time displaying a non-judgmental acceptance of young people’s sexuality.

Second, I focus on the emotional labour that BAC staff performed in their counselling work. Staff had to present themselves as empathetic, caring and reassuring, but they paradoxically also sought to provide heteronormative advice. I argue that this emotional labour was central in BAC staff’s attempt at navigating and smoothing the tensions created by the contradiction between their non-judgmental listening approach to counselling and BAC’s moral conservatism in spreading a vision of committed heterosexual relationships. This emotional labour and the tensions within in it is best illustrated in BAC’s pyschosexual counselling services, which tried to encourage youth sexual pleasure but also taught distinctive gendered sexual roles and heteronormative advice that contributed to pathologising teenage sexual behaviours and desire. This emphasis on sexual pleasure and gendered roles constituted a rupture with the interwar tradition that had only ever addressed the sexual and emotional difficulties of married people, but it could also be viewed as a reshaping of this idea. I contend that, in resorting to an emotionally orientated counselling, BAC members reconfigured for the young the new form of sexual subjectivity that had been in the making since the interwar years, that is, the fact that individuals regarded themselves as sexual beings, which normalised the practice of talking and expressing feelings and anxieties about sex. Indeed, BAC was part of a broader movement that sought to understand fragility in relationship and sexual dysfunctions and also adapted their model to include unmarried and young people. In all, this paper demonstrates that BAC’s counselling work was as much a rupture with the interwar contraceptive counselling tradition—since it operated in a new climate, stressed a non-judgmental listening approach and catered for the young—as it was a continuity of some of the values of the earlier movement in terms of moral conservatism and heteronormative sexual behaviours.

## Counselling young people towards sexual maturity (1964–1988)

The early, stated aims of BAC were ‘the prevention and the mitigation of the suffering caused by unwanted pregnancy and illegal abortion by educating young persons in matter[s] of sex and contraception and developing among them a sense of responsibility in regard to sexual behaviours’ (Brook Advisory Centre 1964). From the start, the centre put to the fore education through counselling by doctors, nurses and counsellors and the idea that young people should become responsible for the choices they made vis-à-vis their sex lives. Consequently, by foregrounding the work of the centre within the framework of counselling and responsibility, BAC members anticipated criticisms of promiscuity and therefore presented a model where responsible behaviours, namely steady relationships that could potentially lead to marriage, appeared as the cornerstone of their work. To a certain extent, this model was a rhetorical device to counter potential criticism. While never affirming it explicitly, this norm was based on the white/middle-class value of committed intimacy. The model did not fundamentally transfigure the principles that informed the FPA. Indeed, by emphasising commitment and heteronormative relationships, the provision of advice to the unmarried was not a departure from the FPA practice but rather should be considered a reconfiguration, with a continuation of the FPA’s general principles and prejudices around class and race.

In a similar vein, Dr Faith Spicer, the first doctor working in BAC in London, defined her work as follows: “If we can show, and I believe we can, that we are helping adolescents to understand themselves, their feelings and their needs, then we are doing [a] valuable job (…) by setting up advice centres of this sort we can help more people towards maturity, (…)” (Spicer 1964, 31). This idea of helping individuals to understand themselves and to reach maturity resonates with Nikolas Rose’s argument that in the second half of the 20th century, experts played a pivotal role in creating a choosing, willing and self-governing self (Rose 1990; Rose 1998). Indeed, BAC recognised the ability of teenagers and young people to make informed decisions, subject to them receiving the ‘right’ type of support and ‘correct’ information. Counselling was therefore perceived by BAC workers as a way of enabling young people to take responsibility for their action and decide for themselves what would be the best sexual behaviours. And clients seemed to have absorbed BAC’s message: as one put it in a survey study carried out in Birmingham in 1968, ‘it was so nice to be treated as a responsible adult’ (Woodward 1970, 88). In the centre, there existed three types of counselling: counselling for contraception; counselling for pregnancy and its outcome and counselling for psychosexual problems. The majority of clients came for birth control advice and supplies, with about 10%–15% attending for pregnancy and sexual counselling. To guide young people towards sexual maturity, BAC counsellors, social workers and doctors defined three essential components of what they perceived as ‘good’ counselling practice: the ability to listen to the client; a non-judgmental attitude and a guarantee of the confidentiality of the services.

### Non-judgmental listening approach

In many reports and committee minutes, listening to the client in a confidential setting retained a central place: Liz Elking, who joined the London Walworth centre as a counsellor in 1974, said that the Brook method was to ‘listen hard, put all the alternatives and try to help the youngsters to reach their own decisions’ (Elking 1974). The roles of staff, in terms of whose responsibility it was to listen to clients and take notes about their needs, differed between regional centres. In Birmingham, from the start, social workers worked alongside doctors and nurses. Indeed, at his or her first appointment, every client in Birmingham was interviewed by a social worker trained in interviewing techniques, whereas in London this interview was conducted by the nurse, who ascertained the reason for the client’s visit and completed the particulars of registration. The interview provided an opportunity for BAC staff to listen to the client’s needs and anxieties and assess the potential need for referral to further counselling services and therapy. A consultation with the doctor followed this first in-depth interview (Birmingham Brook Advisory Centre 1967). In London, at first, social workers had limited power in the centre, since doctors were in charge of the counselling and listened to the clients. But before long, Helen Brook realised that social workers would help give the clinic an unclinical atmosphere, moving away from the ‘doctor-patient relationship’(Brook 1987). She was convinced that social workers were essential for developing a trusted relationship with the clients and allowing the latter to feel listened to. From that point on, social workers had extended responsibility in London clinics. In 1975, Dr Ruth MacGillivray, who was the doctor in charge of the London BAC, announced in the magazine *GP* that counselling was not the exclusive territory of doctors. In her view, nurses and social workers could also be good counsellors provided that they met the essential criteria, namely ‘emphatic listening and the ability to talk simply about anatomy, physiology, sociology and to draw analogies which can be easily understood’ (MacGillivray 1975).

Another paramount element of the way BAC members understood their work was the perceived absence of moral judgement. There was a tension inherent in this, since BAC members were navigating between adopting a pioneering approach to the unmarried via a non-judgmental acceptance of young people’s sexuality and spreading a vision of heterosexual monogamy and sexual responsibility via their heteronormative advice.

In 1969, the task of Brook was defined in terms of education and responsibility and referred to the attitude that individuals working in the centre should adopt: ‘to help young people between the ages of 16 (age of consent) [and] 25 to accept responsibility for their emotional lives without attempting to impose external moral standards’ (Hunter 1969). BAC were first staffed by doctors and nurses trained in family planning practice; their attitude and personality to the young needed to be ‘satisfactory’, meaning tolerant and non-judgmental (Brook Medical Advisory Committee 1964). The doctors usually had added experience in child development and/or psychiatry.

The founder Helen Brook would compare the doctors’ work in Brook centres with that of ‘non-moralising mother-figure doctors’ (Brook 1967). This alleged non-judgmental attitude was particularly well perceived and valued by clients. In a 1967 article published in the *Birmingham Post*, the local newspaper that extensively covered the heated debate preceding the opening of Birmingham BAC, a young white couple shared their experience at BAC. The ‘girl’, aged 21 years, engaged, explained that she had been sleeping with her fiancé since she was 16. She mentioned that she had had ‘a major scare’ and phoned a FPA clinic for help. She was told they could see her, provided that she was getting married in the coming 3 months. Since she was not, they directed her to BAC. The girl explained that she was ‘apprehensive’ before phoning BAC but was amazed by the reaction on the other side of line; the receptionist asked her about the urgency of her needs. The client thought “that was marvellous: they were concerned with your needs, your real needs, and not to sit in judgement on your morals” (Cases and Circumstances 1967).

The ‘motherly figure’ mentioned above by Helen Brook was a recurrent trope deployed by BAC workers when they described their role towards their clients. Time and again, Brook referred to the mother figure: ‘for a young woman the security of a mother figure with the expertise of highly qualified doctors is of untold value when things go wrong, as they so often do, when one is young and experimenting and exploring’ (Brook Advisory Centre). Dr Fay Hutchinson, the clinic doctor in London, in a paper presenting her work at a conference on psychosexual medicine in 1982, would also make this analogy. Referring to a case of a 16-year-old client she said: “When she undressed for examination, you could see the frightened little girl she was, who needed mothering and tenderness. Studying the doctor-patient relationship I realise that “mothering” is not the only role I have, though it is one I feel comfortable with” (Hutchinson 1983, 172).

Nevertheless, this emphasis on the mother figure also had the potential to undermine the autonomy of young people: doctors and counsellors should be understood as another form of authority. Indeed, despite a focus on the non-judgmental attitude, young people were still deemed as a vulnerable category of the population that were in need of advice. BAC doctors and counsellors acted as guides and gatekeepers through young people’s journey towards sexual maturity. Brook doctors and counsellors saw white young middle-class women as responsible young adults who attended the clinic because they were in steady relationships and wanted to protect themselves against unwanted pregnancy. They were perceived as mature enough to make their own decisions. Their behaviour was construed as the ‘norm’ from which other groups deviated. In the first annual report of BAC published in the *Family Planning Journal*, Faith Spicer explained that ‘the greatest proportion of people coming to the centre are young women, quite sure at the time that they have a steady relationship who wish to discuss, often in great detail and sometimes together with their young men, methods of birth control’ (Spicer 1966). These young people were depicted as ‘responsible’. However, any deviation from this model was deemed problematic, and race and class appeared as key elements to define vulnerability. This definition ran against the idea of a non-judgmental approach to teenage sexual needs. Promiscuity, in particular, was pathologised as illustrated by the following quote from the founder of the centre, Helen Brook: “If a girl is promiscuous, you have got to ask why. Promiscuity is a sign of some sort of disturbance. They are the ones who need the most love and the most help” (Brook quoted in Birmingham Sunday Mercury 1966). Promiscuity was usually associated with sexual knowingness on the part of white working-class girls and perceived to be the result of the emotional instability produced by socially deprived backgrounds. Contraceptive counselling therefore functioned as a way of teaching the middle-class values of protected intercourse in a committed relationship and maintaining emotional safety.

For white working-class young women, contraception was described as a means of control; their lack of control over their reproductive bodies condemned them to unwanted pregnancies and abortions. White young working-class women were usually described as having more sexual experience than white young middle-class women, and as such needed more counselling in order to avoid the risks of unprotected intercourse. Indeed, those of lower socioeconomic status, who belonged to classes 4 and 5, were deemed to be at higher risk of unprotected sex since they were ‘socially and emotionally deprived’. These white girls were said to be used by boys as ‘sexual conveniences’ (Crabbe 1978).

Besides class, race was also a key element in defining the vulnerable category that deviated from the white middle-class norm and again partly challenged the vison of a non-judgmental service. Speaking at a conference on ‘Accepting adolescent sexuality’, the first black BAC counsellor Pauline Crabbe, the former Welfare Secretary for the National Council for the Unmarried Mother and Her Child, first black woman magistrate, explained that young black boys, usually from African-Caribbean backgrounds, shared many common problems with socially deprived white teenagers: ‘lack of incentive, lack of opportunity, poor housing, an educational system that failed them, limited job prospects and poor communication within the home’ (Crabbe 1978, 172). This led them to place more importance on sexuality. However, black boys were presented as opposing contraception and encouraging parenthood, while at the same time deemed ‘unprepared to accept the continuing responsibility of fatherhood’. In this sense, they failed to conform to the norm of protected intercourse in a steady relationship. Similarly, assumptions about the apathy of young black women who ‘got themselves pregnant’ also permeated Crabbe’s work. Writing in a 1978 special issue on fertility in adolescence, she flagged up the difficulties faced by young black girls who became pregnant: ‘black girls who have babies in their early teens are especially vulnerable. They are less likely than white girls to fight their way back into education or training for a job and they tend to sink into apathy more quickly and accept a second child because they feel already permanently enslaved by the first birth’ (Crabbe 1987, 173). In drawing on this vocabulary, Crabbe referred to black girls as slaves of their men’s desire. This was a strong wording that distanced their attitudes by sending them back to the time of slavery, and in so doing stressed their differences, therefore othering their behaviours from those of white girls. Crabbe suggested that one of the means to tackle this issue was contraceptive education, in which black adolescent girls would be taught to ‘value their freedom and to show their men the benefits of partnership before they accept responsibility for parenthood’ (Crabbe 1978, 173). While recognising the specific needs of young black girls, these remarks draw on some negative stereotypes about the alleged ‘apathy’ of black girls who became pregnant and the reluctance of black boys to form committed relationships. However, Crabbe also considered black girls to be the ones that made the decisions within the relationship, since they were said to be influenced by the ‘old matriarchal tradition of the West Indies’. Counselling was therefore perceived as a way of teaching white middle-class ‘British’ values around ‘sexual responsibility’ and committed relationships to these young women, so that they could ‘rebel against the prison sentence of early maternity’.

To some extent, BAC members tried to address what they perceived as socio-structural inequalities, since they recognised the additional social challenge that black girls, black boys and white working-class girls faced and devoted energy to finding ways of reaching out to these ‘vulnerable’ young people. Nevertheless, in so doing, they eased the process of assimilation into British norms by conveying standards of good sexual behaviour based on middle-class values, namely protected intercourse in a steady relationship. They thus acted as potential levellers of culturally perceived different sexual behaviours, and this attitude contradicted the alleged non-judgmental approach of their counselling services. For Brook members, maturity and responsibility in sexuality equated committed and loving intimacy.

This tension between non-judgmental counselling and the vision of ‘good’ sexual behaviour was also perceptible in the contraceptive counselling provided by BAC. Since the main goal of the centre was to avoid unwanted pregnancy through the provision of contraception, clients were presented with varied methods of birth control, and the doctors talked the clients through the pros and cons of each method. Some methods were deemed more suitable than others based again on clients’ adherence to the white middle-class norms of committed relationships. The majority of clients came to the centre to ask for the pill. The pill became available on the British market in 1961 on prescription for medical reasons, and at first mainly for married women through private agencies such as FPA clinics. At BAC, at first, doctors seemed to be very reluctant to prescribe the pill for those who did not conform to their moral standard of ‘good sexual behaviour’, namely intercourse occurring in a committed relationship. For instance, at a conference in 1966 aimed at social workers, founder Helen Brook presented several examples of young clients attending the centre; one was refused the pill. The white ‘girl of nearly 18’ came to the centre to ask for the pill. The doctor undertook a long counselling session to find out the reasons why the ‘girl’ wanted the pill. The latter was having an affair with a married man and wanted to be protected against unwanted pregnancy. The doctor refused to prescribe her the pill on the basis that her immaturity was ‘appalling’. The doctor acted as a gatekeeper of morality and judged the girl ‘unsuitable to have the pill’ since she was not in a committed, stable relationship (Brook 1966). After the Family Planning Act of 1967, the pill became available for all women regardless of their marital status, on prescription in FPA clinics and BAC and through general practitioners.

At other times, BAC counsellors seemed to privilege the notion of safety of intercourse over moral considerations about the status of the relationship. Counsellors and doctors qualified unprotected sexual intercourse as risky behaviour. They explained that its prevalence was due to several factors: young people lived ‘in the present and did not plan ahead’ (Brien 2020); they favoured ‘spontaneous behaviour’; there was a lack of acceptance of their sexuality on their own part and on the part of others, especially their parents; their sex education was lacking and they encountered practical difficulties with contraceptive methods (Coles 1978). BAC counsellors and doctors’ role was therefore to mitigate these different aspects by recognising young people as sexual beings when they visited the clinics; they tried to instil in these clients a sense of sexual responsibility through the idea that safe sex was paramount, and explained the practicalities of contraceptive methods. Dr Hutchinson made it clear in a talk in 1978 at a national conference on ‘Accepting adolescent sexuality’ that, for young girls, the pill was not necessarily the better choice since it offered no protection against sexually transmitted diseases. In this regard, condoms were presented as the safest method. No mention was made of whether the young people were in steady relationships or not.

## Emotional labour, navigating values and the model of good sexuality

In the counselling services BAC offered, emotional labour was central in managing the tensions between accepting young people’s sexuality and trying to instil in them a sense of sexual responsibility via the model of committed heterosexual relationships. BAC staff resorted to an emotion-based counselling where clients were encouraged to express their feelings and needs. This emphasis on emotions was not new. From the interwar years onwards, in the field of sexual counselling and marriage relationships, the expression of emotions held a significant role in the understanding of the fragility of relationships, sexual development and sexual dysfunctions. The idea that emotions and authenticity were key to successful relationships were spread by marriage and sexual reformers, the Marriage Guidance Movement, church organisations, the FPA and agony aunts and advice columns in magazines and newspapers, among others (Chettiar 2013; Collins 2006; Harris 2015; Langhamer 2015; Langhamer 2013; Lewis, Clark, and Morgan 1992; Rusterholz 2019). BAC’s work did draw on this tradition and represented a departure from it, since BAC applied it to advising young people, a clear break from the interwar focus on married people. In addition, resorting to emotional counselling was also used as a rhetorical device in BAC’s public discourse. Indeed, BAC based its respectability on providing counselling for emotional problems and relationships, alongside contraception. This emphasis was meant to counter potential criticisms of BAC acting as a ‘contraceptive shop’. In using emotions in this way, BAC staff were trying to find a balance between the progressive radicalism of the clinic in advising young and unmarried women and a form of racial and moral conservatism via the teaching of ‘good’ sexual behaviours.

This balance was first found when counsellors ascertained the right mix and degree of emotions that should be shown; they needed to be adequately empathetic and caring while also maintaining a certain emotional distance and refraining from using emotive language to discuss the client’s situation and needs. This testifies to the ways that the value of non-judgmental counselling was performed through emotional labour. For instance, reflecting on her role in a private phone interview in 2020, the counsellor Joanna Brien, who joined London BAC as a counsellor in the early 1980s, stressed that she was ‘an ally’ (Brien 2020, March 29), and explained that she joined BAC ‘to provide a level of understanding that young people did not have’ since the majority could not talk with their parents or their friends about their sexual life: “Brook wasn’t moralistic, it was sort of saying, yes, this is really enjoyable activity, but you need to do it as safely as you can”. Strikingly, counsellors avoided emotive language in order to let the client reach her own decision without influencing it. Pauline Crabbe explained in an interview in 1987, “I used to school myself by, for instance, not using emotive terms, talking about a pregnancy and not a baby, because that keeps the sort of distance from it. (…) If you press somebody to make a decision by either using emotive terms or anything else it’s not a valid decision” (Crabbe 1987). Indeed, a potential unwanted pregnancy was (and is still) a source of many contradictory emotions, and doctors and counsellors were very concerned with helping the client to disentangle them and identify what would be the best outcome for her, given her own particular circumstances.

Giving the opportunity to young people to express their feelings and to identify and discuss their emotions was central to BAC’s work. In 1982, Dr Fay Hutchinson wrote:

I realise I don’t actually talk to young people very much; most of my effort is directed to trying to get them [to] talk, about themselves, why they’ve come to see me, what they want to do, what’s causing them concern and what they can do to cope with it. Though I may not [be] talking much I am busy observing the patients, trying to assess them, responding to their needs, asking appropriate open-ended questions and giving them time to try and express themselves. (Hutchinson 1983, 172)

Similarly, in an oral history interview I carried out in 2020 with Joan Woodward, a social worker who worked at Brook Birmingham from 1967 to the mid-1980s, she recalled what made Brook so distinctive: “they wanted to offer contraception to young people who would have a chance to come and have a chat, to talk very informally and in a very friendly way with a social worker” (Woodward 2020). BAC workers, then, encouraged informal and friendly discussions and expressing one’s own emotions, a practice that contributed to the stabilisation of sexual subjectivity for young people. For instance, contraceptive counselling was a way to determine whether girls felt pressured to use the pill and to identify the girl’s feelings. As Joan Woodward remembered : when she joined Birmingham BAC in 1967, the pill had become available for all women regardless of marital status. Woodward explained that young girls felt more and more pressurised into having sex with their partner because the partner would say, “get on the pill and we can have sex and you won’t become pregnant” (Woodward 2020). Woodward stressed the difference in attitude between young men and women, since the girls ‘wanted a relationship with somebody, [not just] safe sex with them’. Woodward argued that this situation created tensions and anxieties for young women, revealing the need for counselling. This quotation illustrates the tensions brought about by the availability of safer contraception; this new freedom also functioned as a new form of control for men.

Regarding pregnancy counselling, BAC advisors recommended that time was needed to think about the best decision for the client. However, time was also a sensitive issue, especially for young women, who would sometimes present themselves for help at BAC in an advanced stage of pregnancy. They needed to make a fast decision. As a result, BAC members established a list of sympathetic doctors who would perform an abortion when the waiting time at the National Health Service (NHS) was too long. Dr Ruth Coles, in 1971, explained that she had learnt “how long the NHS pipeline took and the psychological trauma this caused the patient (and me because they were constantly on the phone expecting me to know when this would be done)”. As shown by this quotation, the long process of state-supported abortion created additional anxieties for the client, who had to deal with uncertainty, and the doctor, who was trying to reassure the client while at the same time dealing with the bureaucracy of the NHS.

In addition, BAC counselling also entailed seeking a way to persuade and help under-16 clients to overcome their fears and inform their parents in order to gain abortion approval. Following the 1967 Abortion Act, termination was a possibility, but required the approval of two doctors and, for a minor, a parent. BAC members acted as facilitators for communication between young girls and their parents. The case of ‘Rosie’ (cited in the London Brook Advisory Centre 1980) illustrates this point. Rosie, aged 15 years, came to the centre because she feared she was pregnant, and had a pregnancy test that turned out to be positive. The counsellor first needed to identify Rosie’s feelings about the baby. Doctors and counsellors had a common understanding that if a girl had delayed her visit to the centre, this might point to the ‘woman’s instinctive wish to have a child’. She might not be in the right circumstances to welcome a child, but her emotions told her otherwise, and a delay in seeking help was used as a way to keep the child. However, in the case of Rosie, her main emotion was fear that the responsibility that having a child entailed. Since she was under the age of consent and legally a minor in medical terms, the main issue was the necessity of parental permission to have an abortion; as such, she was paralysed by the idea of announcing her pregnancy. The counsellor had to explain carefully to Rosie that it would soon be too late for an abortion. With the help of the counsellor, Rosie found the courage to tell her mother. In this scenario, then, the role of the counsellor was therefore to provide emotional support for the client as the latter made her decision and faced its consequences, that is, informing her parents. This example is testimony to the emotional struggles clients faced and the essential work of BAC counsellors in helping them navigate their emotions.

In encouraging young people to express their needs and emotions, BAC helped to reshape the new form of sexual subjectivity, advocated since the interwar years where emotions and self-reflection were paramount.

### Psychosexual counselling, pleasure and gendered sexual roles

However, the key area where emotional labour played a central role and where tensions between non-judgmental attitudes and conservative views on sexual relationship were explicit was psychosexual counselling. Sexual counselling had been a focus since the interwar years, but gained in popularity after the World War II, when the emotional stability that depended on the sexual harmony of British citizens was deemed fundamental to the rebuilding of society (Chettiar 2013; Chettiar 2015). Moreover, the development of sexology, with the success of the Kinsey report and its ‘Little Kinsey’ British counterpart (Bingham 2012), put the search for sexual pleasure at the centre of postwar Britain and the stability of relationships. In order to help couples reach sexual satisfaction, many family planning clinics members turned to psychosexual training, which was heavily influenced by psychoanalysis; FPA members were trained by Michael Balint and later the Institute for Psychosexual Medicine (Rusterholz 2020).

Balint was a Hungarian psychoanalyst who worked at the Tavistock Institute of Human Relations in London, one of the key British institutes in psychoanalysis. He devised a method to help general practitioners answer the needs of their patients via training in psychotherapy, as also shown by Robert Irwin in this special issue. Balint was interested in the way that a patient presents his/her illness to the doctor and the latter listens, reassures and suggests treatment. Balint highlighted the dynamic of the relationship between the patient and the doctor, since the way in which the doctor answers the patient in turn shapes the patient’s expectations and manner of articulating his or her needs. As such, the doctor should also be an object of study. Balint believed that a doctor should therefore undergo training to free himself or herself from prejudices (Balint 1957). The training devised by Balint saw doctors exchanging experiences of ongoing cases in small groups and describing their difficulties as frankly as possible, under the supervision of a leader. The dynamic of the group enabled doctors to identify mistakes, blind spots and limitations, allowing a better understanding of their problems and prejudices.

BAC staff underwent this same training, and therefore shared similar views and values. At the London BAC, a consultant psychotherapist attached to the centre held a seminar fortnightly with the doctors. In this seminar, doctors discussed their cases and shared their experiences and difficulties, based, again, on Balint’s technique. The aim of this supervision seminar, explained counsellor Joanna Brien was to work on the assumptions that one held regarding young people’s sexuality and to know what they were, so as to ‘be able to stand back from them’ (Brien 2020). Joanna Brien started working at BAC in the early 1980s after spending a couple of years working for the British Pregnancy Advisory Service—a charity created after the passing of the Abortion Act in 1967 to provide safe, affordable abortions—where she undertook a short period of training in counselling. In Brien’s view, one of the key elements of a successful counselling session was the counsellor’s ‘ability to not impose her own views’. This was perfectly in line with Balint’s training.

While psychosexual counselling did help young people in some ways, with its emphasis on past events and trauma, it nevertheless also contributed to pathologising young people’s sexual behaviours. Psychosexual counselling dealt mainly with anxiety, and as clinic doctor Fay Hutchinson noted, “the younger the client the more areas of anxiety she is likely to have” (Hutchinson 1978). According to Hutchinson, the clients presented with a wide range of psychosexual problems, including lack of orgasm, fear of intercourse, impotence, anxieties related to the body, sexual abuse, same-sex attraction and difficulty with parental relations. Counselling for under-16s was presented by clinic authorities as a way of ‘buying time’ for the girls ‘to continue developing and maturing so that she may make [a] good relationship in the future’.

Over the years, some young men, although still a tiny minority, visited the centres on their own to talk about their fears relating to sexual performance and inadequacy. Orgasmic impairment and premature ejaculation remained the two most common causes for requesting counselling.

In 1967 London, Christie Brown (1967) was hired as a clinical doctor; she started a weekly session for what the clinic called ‘girls with special problems’. The sessions lasted 50 min in order to allow the clients to discuss their anxieties at length. Clients were seen as many times as was necessary to make a diagnosis, or were referred to other bodies for help (Brook Advisory Centre 1968, 40). Christie Brown explained that many unmarried girls needed help with emotional difficulties because they had not fully come to terms with the lives they were living. She stressed again that contradictory attitudes made for a difficult decision for these young people: ‘In a society where parents are likely to regard this action as sinful, conflict is inevitable. These young people have usually seriously thought out the terms of their relationship’ (Christie Brown quoted in Daily Telegraph 1967). From 1970, this openness towards youth sexuality extended beyond the notion of safe sex. She underlined the fact that BAC’s focus was on safe sex and avoiding the negative consequences of unprotected intercourse and sought to ‘help some of them enjoy their sexuality’. Thus, for the first time, the notion of enjoyment and pleasure was finally connected to that of young people. Indeed, while sexual pleasure became increasingly discussed and central to the sexual life of married couples from the interwar years onwards (Rusterholz 2019), and thus became a new field of practice in FPA clinics, associating sexual pleasure with young people was still highly controversial since it was feared it would encourage promiscuity. Sex education in school, when not simply absent, focused mainly on combatting venereal diseases; sexual pleasure was never addressed. While the mass media started to address the issue of sexual pleasure for women in the late 1960s and early 1970s, teenage magazines at that time remained fairly conventional, and did not venture into sexual pleasure (Bingham 2012; McRobbie 1991). BAC was the first charity to take on the challenge of offering practical help with the issue of sexual pleasure for young people. This work might have been potentially influenced by the second-wave feminist focus on women’s sexual pleasure, but this influence was never explicitly acknowledged. However, here again, this progressive idea was undermined by conservative views on the ‘right’ type of relationship and gendered sexual roles. Clients were expected to display maturity, and this was assessed based on their adherence to the white-middle class idea of monogamous, heterosexual, committed relationships. Girls who had multiple partners were described as having troubled personalities, either needing attention or rebelling against their parents (Christie Brown 1974). Boys, as a rule, were never described as being promiscuous. This was largely due to a gendered understanding of sexual development where boys were thought of as having a sexual urge difficult to harness, whereas girls were looking for emotional attachment.

Doctors and social workers, when presenting study cases in diverse publications, invariably applied a gendered framework whereby ideas about femininity, masculinity and gendered sexual behaviours were predominant. These ideas pathologised behaviours that did not fit the model. For instance, Dr Margaret Christie Brown from the London centre applied what she called ‘the Freudian theory of psychosexual development’ to help clients enjoy their sexuality. She considered that past events and childhood relationships could act as an unconscious blockage in the sexual life. For instance, she explained that a proportion of girls were sexually unresponsive because they partially rejected their female role and ‘had identified with their father instead of their mother’ (Christie Brown 1974). She explained that these girls had a history of ‘tomboyishness and were ambivalent in appearance and frequently wore boys’ clothes’. They had a dominant personality and were usually ambitious. Helping them to identify these elements by themselves was thought to be a first step towards sexual adjustment and would offer a way out of pathologised behaviours. Similarly, clinic doctor Ruth MacGillivray had a gendered understanding of young people’s sexual behaviours where ‘boys have tremendous sexual drive’ and girls ‘are more interested in attracting the opposite sex for reasons of prestige and companionship. While enjoying mutual caresses, most do not have the same urge for coitus’ (MacGillivray 1975). This gendered view on sexuality presented male sexuality as inherently animal, uncontrolled and driven by impulse. This implies that girls should be responsible for marshalling young boys’ sexual drive. MacGillivray also encouraged girls to consider orgasm as ‘the excitement felt of seeing a pop idol in concert’ and as an ‘unexpected bonus’. She however enjoined them to find a sexual position where their clitoris could be stimulated since it is the locus of pleasure. She nevertheless reaffirmed that penetration was essential in that it allowed for a more satisfactory coit.

The counsellor Joan Woodward from Birmingham used the method of ‘combining an intellectual understanding of the problem, with where necessary, an increased awareness of the emotional and fantasy aspects through the use of dream material, plus a chance to gain practical experiences free of anxiety through the sensate focus techniques’. For instance, she counselled Jane, who needed help because she had lost ‘her libido’. She presented the case by first emphasising the strict upbringing of Jane, daughter of a vicar, where sex was never mentioned. Jane unconsciously blocked her sexual desire in order to ‘remain the non-sexual child her parents have unwittingly made her’. She talked with Jane about her childhood experience and guided her to recognise ‘conflicted emotions’. At that point, Joan Woodward recommended the programme of ‘sensate focus’, used by the sexologists Masters and Johnson, a series of touching without intercourse in order to arouse pleasurable feelings that lead gradually to genital stimulation and eventually sexual intercourse (Woodward 1977). This diversity of techniques points to the fragmented and volatile nature of sexual counselling, where gendered understandings of sexuality cohabited with more feminist notions of female sexual pleasure. This emphasis on teenagers and young women’s sexual pleasure was new in the context of British society in the 1970s, and constituted a continuity with the work done in FPA clinic to encourage sexual pleasure.

## Conclusion

In the early 1960s, young people’s sexual behaviours created anxieties triggered by an increase in premarital sexuality. In spite of growing debates about the need to tackle this trend, the main sexual health charities, such as the FPA, limited their scope to married individuals. BAC was therefore the first service provider to openly focus on young people. The counselling offered by BAC was meant to educate young people towards sexual maturity and encourage them to take responsibility for their sexual lives. In so doing, BAC did not represent a departure from the values favoured by the interwar tradition of contraceptive advice as exemplified by the FPA, but instead readapted these values for young people. The counselling practices provided by BAC were required to follow the ideal of a non-judgmental listening approach to young people’s sexual needs. However, this ideal was undermined by a moral conservatism that favoured heteronormative sexual behaviours based on the white middle-class model of protected intercourse in a steady, loving heterosexual relationship. Indeed, race and class had a powerful impact on the classification of people into vulnerable categories in need of counselling. Indeed, prejudices were not always conscious, and though attention paid to one particular group might have emerged from empathetic considerations for the reproductive outcomes of individuals from that group, it nevertheless remained the case that stereotypes and generalisations underpinned these considerations. Counselling might therefore have been used as a way of teaching the white middle-class value of commitment through the ideas of love and sexual responsibility.

This paper also shows that counsellors encouraged young people to express their emotions, contributing to the stabilisation of a new form of sexual subjectivity where talking about feelings and sexual behaviours became normalised for young people. This emotional labour also helped to manage the tensions created by BAC’s antagonist aims. Finally, the paper looks at psychosexual counselling in detail and shows again that tensions were present. Young people were recognised as sexual agents who could enjoy their sexuality. This was radical. Indeed, until this point, the idea of helping married couples to achieve sexual pleasure had been gaining momentum in FPA clinics, but the idea had never been applied to young people due to fears that it would encourage promiscuity. However, while sexual pleasure was addressed, providing a novel element, the idea of promiscuity limited the sexual freedom allowed to young girls. In addition, sexual counselling drew on a gendered understanding of sexual behaviours, where young men were thought of having greater sexual drive and young women were in search of emotional attachment; this framework, to a certain extent, pathologised young people’s sexual behaviours when they deviated from these gendered sexual roles.

BAC members helped to establish a new vision of the teenage sexual self, where responsibility, commitment and protection functioned together. This emphasis on emotionally committed relationships was part of a broader trend in counselling work, where emotional maturity was perceived as the cornerstone of British society.

## Data Availability

Data sharing not applicable as no datasets generated and/or analysed for this study. All the archival materials are freely available from the Wellcome Library, SA/BRO, in London. The four oral history interviews conducted by the author with former counsellors are not available to consult. Former counsellors have only granted the author authorisation to use their interview.
